# Harms of social regulation on the development of maternal identity among sex workers

**DOI:** 10.3389/fpsyt.2026.1771867

**Published:** 2026-03-19

**Authors:** Regan A. Moss, Aurelie Athan, Rebecca L. Fix, Lisa Sholomon, Silvia Vilches, Kelli S. Hall

**Affiliations:** 1 Social, Behavioral, Population Health Sciences, Tulane University School of Public Health and Tropical Medicine, New Orleans, LA, United States; 2 Department of Counseling & Clinical Psychology, Columbia University, New York, NY, United States; 3 Bloomberg School of Public Health, Johns Hopkins University, Baltimore, MD, United States; 4 Human Development and Family Sciences, Auburn University, Auburn, AL, United States

**Keywords:** carcerality, maternal identity, maternal mental health, matrescence, sex work

## Abstract

**Introduction:**

The maternal identity development, known as matrescence, of people engaged in sex work has been largely overlooked. Legal system involvement and social discrimination may have disruptive impacts on their developmental experience of becoming a mother. The EMERGE Study (Exploring Maternal Experiences and Reproductive Identities in the Sexual Gig Economy) explored factors such as maternal identity and maternal mental health needs among sex-working moms across the US.

**Methods:**

Six former and current sex workers and victims of commercial sexual exploitation who identified as mothers or maternal figures (hereafter referred to as sex-working moms, MSW) participated in semi-structured, in-depth individual interviews.

**Result:**

These MSW detailed their experiences with motherhood and how stigma, discrimination, family policing, incarceration, and surveillance negatively impacted their mental health, maternal identity, and sense of self as a mother. The results demonstrate the harms that the legal system has on sex workers’ maternal psychological wellbeing as well as their identity as mothers.

**Discussion:**

Recommendations include amplifying positive formations of maternal identity which may improve maternal mental health outcomes among sex-working moms.

## Introduction

Many sex workers are parents (1) and describe great joy and pride in their identity as mothers (2, 3). However, maternal psychological experience among people who are mothers and in sex work (MSW) have not yet been thoroughly explored in the current scholarship (4). Research into the unique challenges faced by MSW, and the potential impact of how sex work is perceived on maternal mental health outcomes offers a promising line of inquiry. Furthermore, viewing MSW through a human developmental lens, one that affirms the normative transition to motherhood irrespective of their line of work, allows an overlooked aspect of their experience to be foregrounded, namely how they perceive their own dignity and belonging in the role of mother. Unfortunately, many sex workers report negative experiences with the legal and carceral systems, such as casting doubt about whether they are deemed a “good” or “deserving” mother because of their income source. This may place them at greater risk for a disrupted maternal identity formation by casting doubt on their legitimacy or right to be recognized as mothers. The purpose of this paper is to describe the experience of matrescence, or the developmental transition to motherhood (5), among MSW, particularly how discriminatory socio-legal interventions may have the unintended consequence of contributing to poor maternal mental health by fostering internalized stigma about their adequacy that undermines their emerging maternal identity and self-confidence.

### Mothers in sex work

Previous studies highlight the complex intersection of motherhood and sex work: the systemic injustices, challenges, stigmas, and structural barriers faced by women who live both roles simultaneously. Much of existing work has focused on low-income, street-involved workers (i.e., “prostitutes”). Dewey (2015) describes the challenges low-income MSW face when working to provide for their children in a culturally acceptable manner. Dewey found that, in an effort to “combat the marginalizing process at work that label them as ‘bad’ mothers”, many MSW felt pressured to perform motherhood in ways that aligned with more socially acceptable norms (6).

### Stigmas among moms in sex work

While stigma surrounding motherhood may inform how MSW mother, in the active sense, stigma may also impact legal parenting rights and maternal mental health. Considerable sexist stereotypes of substance use, neglect, and stability surrounding sex work and narratives of normative motherhood are embedded into many legal landscapes (7). In addition, the heightened surveillance of sex workers who are mothers can lead to forced child removal (7).

Sex work stigma may manifest in multiple forms for MSW. First, MSW could experience internalized stigma of sex work stigma, which can impact maternal health care utilization and delay help-seeking behaviors, contributing to adverse mental health outcomes (8). Derived in part from social stigma, criminalization of sex work also impacts access to prenatal and postpartum care for MSW (8, 9). As an example, healthcare providers may discriminate against MSW, leading to violations of patient confidentiality, assumptions of being ‘infected’ [with HIV], provider-facilitated prejudice that lead to delays accessing care during labor while in healthcare settings, and forms of psycho affective obstetric violence (8, 10, 11) MSW also report social exclusion or isolation in healthcare settings from other mothers (8, 10, 11). In response to and out of fear of discrimination and/or arrest, some MSW may avoid disclosing their occupation to healthcare providers, which can complicate the provision of care, as they cannot provide medically accurate and responsive recommendations (11). When sex work stigmas are pronounced in the clinic, such as through provider discrimination, MSW may avoid seeking healthcare entirely. This may be a strategy to avoid facing criminalization or family policing via Child Protective Services (CPS), if their occupation is reported by healthcare providers. Fears held by MSW about hyper-surveillance by mandated reporters and CPS investigators are rooted in historical precedent, whereby children of sex workers are often removed from their care and their occupational status is used as a means to criminalize parenthood (6, 7). For instance, MSW have reported encounters with the police while working, which have led to their children being relocated into foster care upon conviction (13). Discriminant policing practices or punitive policing towards sex workers, and subsequent consequences on child custody, have led workers to avoid seeking legal support for wage theft and/or client abuse, in fear of retaliation from the police (6, 7, 14, 15).

### Mental health implications

In a recent review, Kalinowski et al. (16) found that the prevalence of mental health diagnoses (e.g., depression, anxiety, post-traumatic stress disorder (PTSD), suicidality) among sex workers is highly variable which may be due to the vast variety of occupations within the sex industry. Interestingly, while pregnancy and early infant loss increase the risk of depression, parenthood, on the other hand, acted as a protective factor (Kalinowski et al. (16) for suicidality among MSW.

Traumatic or adverse reproductive and parenting events are directly associated with poor maternal mental health outcomes and indirectly connected to distress, suicidality, and stress through other stressors (e.g., violence, unsafe working conditions, child custody challenges). Fear of CPS involvement is associated with barriers to accessing healthcare during pregnancy, which can extend to seeking psychological counseling (17). MSW present with significantly elevated rates of mental health problems (18) and given that people living with mental illness are also indiscriminately targeted by CPS (19), the medical-legal landscape is further complicated for MSW.

Mental health challenges for MSW may therefore be cyclical: involvement with CPS is associated with depression, anxiety, and stress due to factors such as perceived and actualized stigmas, loss of parenting decisions, and the trauma associated with child removal (20). Thus, discriminatory family policing due to sex work status or mental health stigma is compounding. Policing and criminalization can impact the maternal mental health and wellbeing of MSW during the early years of their child’s life as it may lead to internalized stigmas or the embodiment of negative narratives about their own mothers.

### Legal impacts on maternal identity development

The specific impact of criminalization and related legal-system practices on the maternal identity development among MSW remains insufficiently examined, despite clear evidence that legal stigma and surveillance shape maternal mental health more broadly. Within the intersection of sex work stigma and motherhood, the developmental transition of motherhood, also known as *matrescence*, offers a much-needed lens for understanding how external forces can alter the internal psychological work of becoming a mother. Matrescence, like its counterpart adolescence, is a human developmental window involving significant biological, psychological, and social changes (21). When legal interventions introduce threat or instability during this period, and beyond, they can disrupt the formation of a coherent maternal identity, heighten stress, and undermine a mother’s ability to integrate the role of sex worker and mother into a cohesive sense of self. These disruptions may contribute to heightened stress, identity confusion, contaminated narratives about themselves as mother, and diminished confidence all which may have downstream mental health consequences not only for the mother but also the development of a secure bond with their child. In addition, narratives of *normative motherhood* embedded in social institutions and cultural discourse (22), can create existential barriers for MSW by defining motherhood in ways that exclude or delegitimize them. When mothers are denied recognition as full participants in the maternal role, whether through moral judgment, structural discrimination, or legal sanctions, their developmental work of matrescence is constrained. Under such circumstances, mothers may have limited opportunities to enact mothering practices, cultivate meaningful relationships with their children, or challenge the mischaracterizations of their parenting that they are pressured to accept. The inability to hold both identities, mother and sex worker, within a stable sense of self represents a core conflict that must be resolved.

These internal conflicts can be both subjective and existential but also objective and logistical. For instance, for MSW who experience incarceration and are subsequently removed from their children (physically and/or legally), experience interruptions in the day-to-day relational processes that support maternal-child attachment. Although no studies to date have specifically examined the experiences of previously incarcerated MSW as they navigate motherhood upon reentry, a robust body of literature documents the pervasive harms of incarceration on maternal self-efficacy, and the narratives imposed on mothers involved with the criminal legal system (23, 24). Existing scholarship does not sufficiently emphasize how such sociolegal intervention reverberates beyond immediate practical consequences to shape mothers’ developmental trajectories, sense of self, and long-term mental health.

### Study purpose

The purpose of the current study was to elucidate experiences of matrescence among MSW, and explore how family policing, surveillance, and incarceration impact maternal identity and matrescence, and consequently, maternal mental health. Specifically, our guiding research questions sought to better understand disruptions during the developmental transition to motherhood among MSW which were legal in nature: *1) How does the legal system interact with MSW during matrescence,* and *2) What is the impact of the legal system during these encounters on sex-working mom’s sense of self and maternal identity?*

## Methods

Data for the current study were derived from the first phase of EMERGE: *Exploring Maternal and Reproductive Identities in the Sexual Gig Economy* project, an ongoing study exploring identity development among MSW in the US. The focus of the EMERGE study is on cognitive models for maternal development, or maternal developmental frameworks. MSW refers to mothers in sex work, maternal figures in sex work, and mothers who have been commercially sexually exploited in the US.

The study received approval from the [Blinded] Institutional Review Board. The interview protocol was developed and piloted between fall 2023 and the spring of 2024. The interview protocol for the EMERGE study was semi-structured and focused on reproductive identity (i.e., strength and centrality of orientation towards parenthood or non-parenthood) (25), matrescence, and experiences of motherhood from preconception to the present day (e.g., postpartum to young/middle life motherhood) informed by Athan (21). Questions included, *“What’s it like to think of yourself as a mother?”* and *“What is the emotional impact of motherhood for you?”* In addition, participants completed a brief sociodemographic survey. Following the pilot in 2023, the protocol was refined to more aptly capture maternal developmental frameworks for the full EMERGE project.

### Recruitment

Participants were recruited by circulating study flyers to community-based organizations that support the health rights and needs of female sex workers and sexual exploitation victims in the US. Individuals were eligible to participate if they identified as MSW, were 18 years of age or older, and were able to participate in an interview conducted in English. Consent was gathered verbally and MSW who completed interviews received $50 e-gift card remuneration for participation. Interviews began in the fall of 2023 and, following revision of the protocol, were re-started in fall of 2024. The current analysis utilizes all data gathered before January 2025.

### Population

Six (*N =* 6) mothers participated; one participant did not complete the demographics or psychometrics survey. Participants variously identified as mothers through childbirth, marriage, or adoption, highlighting a range of pathways and experiences of motherhood. Three participants had one child, two participants had two children, and one participant had five children. Three participants had children aged 3 or under, and two had children who were older than the age of 3. One participant was pregnant at the time of her interview. Most (*n =* 4) participants identified their relationship status as single. Two participants described themselves as biracial (e.g., Black/African American and white); one identified as Black/African American; two participants were white. All five women identified as heterosexual, cisgender women and were 30–51 years old.

All participants identified with the term “sex worker,” however, the types of engagement in sex work varied from full in-person services to adult content creation for synchronous and asynchronous consumption. Participants included one former sex worker, three current workers, and two MSW who identified as sex trafficking survivors. Frequency of engagement in sex work among those currently working ranged from regular to occasional or spontaneous. Some women considered it a full-time job, whereas other participants described it as a side hustle or a way to earn supplemental income.

### Data collection

The qualitative interview guide was drafted by the first and second author to explore matrescence among sex-working moms. The interview guide was adapted from previous interview guides that have been used with mothers who are not sex workers. Domains covered domains of matrescence – spiritual wellbeing, psychological wellbeing, economic wellbeing, biological wellbeing, etc. Examples of questions would be, *What brought you to become a mother? Did you have a desire to become a mother? What’s it like to think of yourself as a mother? What is the emotional impact of motherhood for you? What are you learning as a result of motherhood? How do you experience motherhood?*

Interviews were recorded via Zoom video-conferencing technology. Participants reserved the right to turn off their video at any point during the interview. Audio files from each interview were recorded, transcribed, and stored in a private, secure cloud-based platform accessible only to the research team. Video recordings (which were automatically recorded by Zoom) were deleted after interviews. Participants were given an identification number (e.g., EMERGE #001-006) to shield identity. Upon request, participants could request a copy of their interview transcript.

### Interview

We are aware that members of the MSW population have historically been and continue to be mistreated in academic settings, and thus have a high level of distrust. In response, we made concerted efforts to build rapport with interviews to ensure that participants have strong trust in the research, comfort in participation, and ongoing opportunities to ask questions before consenting to participate. Participants were probed to share stories which informed their outlook on motherhood and external forces that shaped their perspectives on being a mother.

Interviews took place on Zoom or via phone call. Participants were given the opportunity to speak with and ask questions to the research team prior to the interview. Prior to each individual interview, the interviewer emailed a copy of the Consent Letter to each participant and during each interview read the study’s Consent Letter aloud and asked if participants had any questions. Participants then provided verbal consent to proceed with the interview.

### Survey

After completing each interview, the interviewer sent participants an online, 10-minute Qualtrics demographic and mental health survey which requested additional information about maternal mental health. For the current analysis, we utilized the demographics portion of the survey to contextualize the qualitative responses of each participant.

### Analysis

Thematic content analysis was used to identify how policing, incarceration, and surveillance – as products of criminalization and stigma – disrupted normative developmental arcs among MSW during intense periods of parenting. Codes were applied deductively, through existing autobiographical accounts from MSW highlighting the sociolegal interventions they were exposed to. Codes developed included: *prison, jail, police, policing, violence, legal protections, child protective services, court, custody, rehabilitation, treatment, healthcare providers, stigma, judgement, discrimination, incarceration, banking.* The lead author coded manuscripts and coding was then discussed with the second author to ensure validity with the framework of matrescence.

Coding revealed how the legal system disrupted maternal identity formation and influenced defining the self as a mother, leading to a pilot analysis of the first interviews. To promote trustworthiness (specifically credibility), the lead author developed and refined the final themes through discussions with maternal psychologists (coauthors). Survey data on demographics were used to characterize the current sample.

## Results

Four themes emerged from the coding, which describe legal and structural barriers to matrescence. These include: 1) Questioning Maternal Competence; 2) Policing Mothering Acts/Performance; 3) Guilt and Psychologically Identifying as a Mother; 4) Who Are We? Bonding with Other MSW.

### Questioning maternal competence

Participants reported experiencing discrimination in healthcare settings, attributing it to provider-based sex work stigma. One participant described being labeled high-risk due to her work, which undermined her sense of control and agency in caring for her child.

“I remember one time when I had my son, he ended up being thrush on his tongue. I was sitting in the hospital. My baby was a newborn. He had thrush. The lady came in and she says, “Well, do you have HIV?” “Well, haven’t you looked at my papers? No, I don’t. Why are you asking me that when it’s clearly documented.’” [001]

This participant recounted how she was fulfilling all of the expectations and responsibilities of motherhood by taking the child to the doctor, but felt that her mothering acts were not “enough” for the healthcare staff, who judged her competence on the basis of her status as a sex worker, and made assumptions about her health rather than responding appropriately to her child’s needs. The staff questioned whether or not her child actually had thrush and instead, proceeded to question her about her HIV status.

Outside of primary clinical healthcare settings, participants also could feel judged by community care providers. A participant described an eight-month struggle trying to obtain healthcare for prenatal care and labor, which was not available through her employer due to the illegality of the industry. When accessing basic prenatal care through a mobile unit, she experienced extreme judgement where providers questioned if she should mother and suggested she gave her child up for adoption. As her identity as a mother was beginning to form, others questioned if she deserved this role.

These paralleled experiences seeking support from a food bank:

The women were very judgmental, and I didn’t know that was going to happen. So they were asking me if I would accept Jesus and all this stuff. And I just told them yes, because I like felt like this is going to make them like, at least be nicer to me, because they’re trying to, like, tell me that I needed to get like, give my child up for adoption because I was in a financial position to not have a child, which I was super, super upset about. And then I think I went to, like, a food bank too, and they said something along the similar lines, and I cried. I cried in front of all the people; I didn’t care. It was just so hurtful, because I’m like, just because I’m having a hard time. [003]

This messaging was hurtful, and began to impact her early identity as a mother. While pregnant, she was performing motherhood; however, she felt discriminated against – from both clinical and community spaces – right from the start. Similarly to other participants, despite believing that she was performing motherhood appropriately (e.g., seeking prenatal care, providing food), this participation reported feeling discriminated against because of her economic status and occupational position, such that others suggested that instead, she not *parent* (in the active sense) at all. She felt that others were discounting or belittling her role as mother.

### Policing mothering acts/performance

Family policing (i.e., encounters from CPS) was the most present driver of mental health needs, including disruptions to maternal identity and sense of self as a maternal figure. Two participants had direct, years-long encounters with CPS. One participant described the deep traumas that she and her children faced surrounding CPS, recounting a period of time where she was working on her sobriety. While her son was in a foster home, she had visitations with him.

Then my son, he wanted to be near me. He was only four years old. I tried everything to keep him and tried to do … No, not *try*. I *was* doing the right thing. The next visit, I went and got my son, and I was washing his shoes, and we were watching TV. Then next thing I know, I get a knock on the door, and she comes to the door, or I open the door, and she says, “I’m coming to take your son. Your visits are terminated, and you will be gone back to court.” And she just grabbed him. He didn’t have his shoes on. He started crying, and he cried all the way across the street. It was so devastating because he didn’t know. It was almost like retraumatizing [ … ] He was devastated. He cried all the way to that car, and he was screaming. And I fell to the floor, and I called my outpatient counselor. [001]

Meeting norms established for affluent mothers was especially challenging, as criminalization led to a lack of legal protections. The lack of legal protections for sex workers exposed MSW to unique economic vulnerabilities, increasing risk for mental health problems and need for mental health support. A participant shared a story of a colleague from a strip club who, following a Cesarean (C) section, was unable to take time off from work to heal or bond with her children. She described that her friend was also being sexually exploited:

But then that was like, borderline, is this guy her pimp? I’m just being honest, because that relationship didn’t seem like a normal relationship. It seemed like that girl that had her C section and came back to work. I don’t think she had a normal relationship. Unfortunately, I just I can’t, I can’t. I cannot accept that she did. I cannot see a man that is a loving man that would be okay with that. [003]

Exposure to violence and limited access to legal protections made it difficult for participants in tumultuous or violent relationships to seek prenatal care. One participant described relapsing and having difficulties accessing postpartum mental health support due to violence in her relationship:

From there, things escalated as far as my addiction, my marriage was dissolving, and it was very violent at the time. And I ended up going back home [ … ] to my family. So, I didn’t have any postpartum [resources] at all. [001]

A violent marriage on top of an escalating addiction exacerbated adverse mental health outcomes. The participant did not have access to care to support postpartum mental health needs.

Due to criminalization, MSW could not obtain medical leave, health insurance, and maternity leave, despite having employment. This participant continued her story, describing her coworker’s return to work immediately after being released from the hospital. “I think she got off of work and she went and had her C-section, and then [ … ] as soon as they let her out of the hospital, she came back to work. She showed me her C-section scar. It was fresh” [003]. This MSW was not afforded the time away from work to bond and heal with her child.

This participant went on to describe the long hours she and other MSW worked and their inability to access social policy support through work (such as childcare) due to criminalization.

And then after they got off work, they could take a shower, take a quick nap, and then they had to take their kid to school. Or if it was the weekend – because usually those are the better days – [ … ] they have to be awake with their kids, and then they would put their kids to sleep and come back to work. Some of those women would be so tired, so when it would be like, slow there, they would be taking naps, like in the locker room, on the floor, under the bench, even sometimes out in the club, they would fall asleep in the chairs. [003]

The constant juggle between motherhood and sex work leave MSW feeling exhausted. After giving birth, the lack of legal protections tied to employment led to a loss of health insurance. A participant described how she could no longer access healthcare for her needs: “I think it is two months after you have the baby, they do your postpartum checkup. And then they were like, “Congratulations! You have now been kicked off the insurance. But it’s okay – your baby is still on there!” [003].

### Guilt and psychologically identifying as a mother

This participant described the traumas she had due to CPS encounters and the experience of feeling out of control, even though she did all the “right thing[s].” As with other participating moms, she recounts the physical acts she was doing for her child (e.g., washing his shoes), things that were markers of mothering. The distress due to the loss of custody (removal of visitation rights) of her son nearly caused a substance use relapse. For this participant, it felt impossible to perform motherhood in a way that was acceptable to the state. Decades later, she was stuck in grief and guilt and had not yet acknowledged the damage and trauma she had endured. She recalls his screams as he was carried away by the CPS worker, with no explanation as to what was happening or how his life would soon change.

Another participant (who was sexually exploited by an ex-boyfriend) lost custody of her child for several years due to pregnancy criminalization (i.e., criminalization of substance use during pregnancy). Through sexually exploitative circumstances, she developed an addiction facilitated through her boyfriend who was her trafficker/abuser.

Well, like I was in addiction [ … ] Well, after I had her, I was sober the whole time, but I just got back in her life, like about two years ago. But [before then], I was in addiction. I was on crack cocaine for a while, so, like, I just learned, like, that whole time that I lost, like, it was a lot of time. [005]

Though the participant’s baby was able to receive support for withdrawals, the participant herself was unable to access care. Discontinuity in care led to additional time she spent navigating an addiction. The lack of treatment led to lost time to bond with her child. Due to the gap in care, she felt continuously exploited, and in turn had to navigate postpartum depression without any support.

“It was, it was bad, like, I was very depressed, you know, just it was like, you know, it wasn’t like I was very depressed, like, it was just very hard, you know, trying to go to sleep and stuff, but like, I, I was like, after, like, I was already in my baby’s life until, like, she turned five, so I really don’t know, like, for me, it was bad because I was on drugs and I was in the streets. So, I don’t know if that’s considered postpartum.” [005]

This participant even questioned if her experiences during the postpartum truly were considered the postpartum. The participant questioned whether her subjective experience of the postpartum period, a critical period of motherhood, was ‘legitimate’ and whether or not her unique experience of being in the street (i.e., homeless) and using substances ‘counted’ as it was not the same as how postpartum had been portrayed to her by other mothers. The participant carried shame around her substance use habits.

All participants who experienced pregnancy criminalization – which often extended into the years following birth – were simultaneously experiencing domestic violence and/or commercial sexual exploitation. Stigmatization from both clinical providers, however, prevented them from receiving access to mental health support. Thus, without adequate support, mental health needs from child custody loss, domestic violence, and substance use grew.

### Who are we? Disrupted bonding with other MSW

CPS-interventions disrupted MSWs’ ability to bond with other workers over motherhood and share their maternal roles with one another. A participant recounted how several of her peers did not share any information about their children because they might ‘lose’ them through the system of foster care or family policing.

A lot of workers don’t share that information only because, if it does get back to the wrong people, then they can call CPS or DFS [Department of Family Services] or the state or different things like that, and take a chance of losing their kids through the system. That’s probably why a lot of mothers you know don’t really talk about their kids, only because if it gets back to the wrong individuals, then you’re taking a chance of your whole world crashing from losing your kids, losing I mean, a whole lot if the state gets involved. [002]

For this participant, motherhood was central to who she was. However, she did not get to present that core part of herself to others. She felt the state had the unfettered power to disrupt the core parts of who she was. She did not have the space to find a sense of belonging as a mother and build social connection among other moms.

Fear of losing children also deterred people from seeking healthcare for a variety of needs, beyond postpartum mental health support: “Some people are scared to go to the hospital because they don’t know, you know, they don’t know what they’re gonna [do to] that kid at the hospital” [005]

## Discussion

Matrescence has not been robustly examined among mothers whose experiences deviate from the dominant cultural narratives of *normative* mother such as MSWs. Yet expanding our understanding of maternal identity formation across diverse pathways to motherhood is essential for ensuring that maternal mental health frameworks are inclusive and applied to all mothers, not just those deemed deserving by dominant motherhood discourses of *normative* motherhood. This study contributes to this gap by investigating how MSW interpret persistent disruptions to parenting and how legal-system involvement becomes a significant site where maternal identity is questioned, destabilized, and reshaped. Further, this study adds to the discussion of “bad mother myths” and dominant morality discourses surrounding motherhood (26, 27), as described within the substance use and pregnancy literature.

Findings illustrate that MSWs encounter a distinct pattern of disruption – legal, social, and existential – that shape how they experience matrescence and how they come to recognize themselves as mothers. Whereas existing scholarship documents that many MSWs feel their legal rights to parent are rejected, participants in this study described a deeper psychological impact: the erosion of their right to psychologically identify as a mother.

Participants consistently emphasized how legal systems shape the inner work of becoming a mother, especially in moments of heightened vulnerability such as pregnancy, early parenting, or periods of isolation or single-parenting. Continual disruptions prevented mothers from developing continuity in their relationships with their children, engaging in the caregiving acts they viewed as central to their maternal role, or resolving the negative stereotypes imposed upon them. This study also highlights the reciprocal relationship between maternal behavior and maternal identity and sense of self: legal restrictions and structural precarity (e.g., lack of labor rights, economic deprivation) limited mothers’ ability to enact desired caregiving behaviors (e.g., purchasing shoes for a child, accessing nutritious food during pregnancy). These findings highlight how direct and indirect forces – such as CPS involvement and structural determinants of health – constitute forms of *structural violence* that target mothers’ agency, narrow the caregiving practices available to them, and diminish opportunities to sustain a positive maternal identity.

Prior research shows that “dominant mothering discourses” can generate guilt and unattainable expectations (28) For MSWs, these dominant ideals are exceptionally difficult to meet without material resources, and navigating them requires mothers to negotiate agency, finding opportunities to reassert their “maternal autonomy” by trying to make decisions for their children (29). Social cohesion and connection with other mothers with shared experiences can lead to a reduced risk in internalizing the ‘perfect’ mother mythology. A central theme of this study was the extent to which legal practices, and particularly those tied to family monitoring systems (CPS), produce guilt and shame, identity instability, and lack of pursuit of maternal mental healthcare. Participants described questioning not only their mothering behaviors (e.g., washing shoes, taking a child to the doctor, gathering groceries) but their very legitimacy as mothers, echoing the identity erosion documented in prior work and questioning the legitimacy of their motherhood and their mothering acts. Elsdon et al. (29) for example, describes this questioning as a reduction in confidence in decision-making, which places approval for mothering acts externally instead of within the self. For some MSW, CPS led to physical or legal separations from their children, creating long-lasting effects lingering for decades after the original encounters – leaving some mothers with the sense that their children did not even belong to them (30). In several instances, the loss of control in providing for their child coupled with being denied their motherhood (or status as a mother) by the state led to continued behavioral health habits (e.g., substance use) that mothers otherwise wanted to stop or seek treatment for, and also furthered experiences of sexual exploitation. Even though participants in this study were not imprisoned, their experiences mirrored those of incarcerated mothers (e.g., child custody loss, feeling othered in their mothering) due to this “professional surveillance”. Garcia (31) emphasizes the need for resources – such as child-reunification programs – that reflect the diverse realities of mothers and the many forms motherhood can take, rather than imposing narrow, socially constructed definitions of what a mother should be. As Garcia notes, failing to do so creates additional obstacles for mothers, a pattern reflected in our participants’ experiences seeking healthcare and food-bank support.

Participants also experienced pregnancy criminalization, and the policing of substance use while pregnant highlighting a broader legal landscape in which the State aggressively scrutinized their motherhood while failing to protect them from violence, exploitation, or partner abuse. Despite exposure to significant harm, participants rarely received victim services, trauma-informed care, or substance-use treatment during the perinatal period. This combination of surveillance without support left many mothers feeling invisible, undeserving of care, and structurally abandoned without access to women-centered models of care in their community (32, 33). Many of our participants directly experienced or witnessed violence by clients, traffickers, or intimate partners. Finally, although participants did not report policing within healthcare settings, they experienced discrimination that threatened their already vulnerable sense of maternal self. Participants were questioned about their legitimacy while engaging in everyday acts of mothering such as buying food, accessing healthcare, or seeking support for their children. These experiences reveal that MSW are not merely judged on their ability to *mother* but on their right to be *someone who is a mother*, a foundational prerequisite for healthy maternal identity formation. For many in this study, motherhood was a central, cherished dimension of the self; legal and structural forces that delegitimized this identity were profoundly disruptive and psychologically injurious.

### Implications

Despite the state-based and state-perpetuated discrimination participants encountered, they also expressed profound pride in their identities as mothers – an established finding within prior research (2, 3, 29). Services that build on this sense of pride, particularly sex worker-led community care, can amplify protective factors and buffer the harms of sociolegal intervention and stigma. Supporting mothers’ developing identities may strengthen their sense of self and enhance coping capacities in the face of structural stigma. The nonprofit and social services sectors play a critical role in cultivating social cohesion and peer support among current and former sex workers. Developing spaces that honor MSWs pride in their motherhood can promote maternal agency and resilience. Peer support groups, community programming, and identity-affirming activities offer opportunities for mothers to share positive experiences, name sources of strength, and counter stigmatizing narratives by reinforcing their growth-producing aspects of their becoming a mother. Within healthcare settings, there are considerable opportunities to improve practice. Participants described discrimination across the reproductive continuum from prenatal care, through labor, and into postpartum and early infancy. Clinics can implement provider bias training to reduce stigma, improve trust, and ensure that MSW receive dignifying, affirming maternal healthcare. Many participants faced lasting harms from CPS involvement, and ensuring access to income benefits, stable housing, and mental health services is thus important to restoring MSWs’ reproductive rights and parental wellbeing.

Legal protections remain largely inaccessible to MSW. Participants described the physical and emotional strain of long sex-working hours, domestic responsibilities, and caregiving without access to childcare, maternity leave or family leave due to the criminalized nature of their work. These burdens further impeded their ability to manage their mental health needs, especially without co-caregivers or employer-sponsored benefits available to other working parents. This research can inform ongoing scholarly debates about the legal status of sex work by illustrating how criminalized contexts limit MSWs’ access to basic workplace protections, such as postpartum insurance, maternity or family leave, and sick leave, that are essential for supporting maternal health. A matrescence framework highlights how discriminatory legal structures undermine the developmental processes that support maternal wellbeing, suggesting that any policy reforms addressing sex work should account for the conditions required for caregiving, healthcare access, and parenting with social support.

As Kalinowski et al. (16) argue, addressing the mental health needs of MSW will require “policy reforms that consider the complex interplay of various factors affecting sex workers.” An adequate response must extend this line of thinking often used by those supporting decriminalization toward one promoting the full humanity and dignity of sex-working mothers. This includes addressing the inequitable distributions of power and resources that lead to the policing, surveillance and incarceration of MSW. Such upstream efforts are crucial to supporting the maternal mental health and developmental wellbeing of MSW and their families.

This study highlights opportunities for the larger community of researchers to better understand how encounters with the legal system shape both mental health and maternal identity among MSW. By broadening our conceptualizations beyond pathologization and into consideration of human developmental science, we can deepen our understanding of how legal interventions may alter the trajectory for a mother’s identity formation. Such work is essential for acknowledging the long-term consequences of punitive healthcare and legal systems on maternal wellbeing and family stability.

### Limitations

The response size is currently not large enough to make statements about causality between stigma, identity, and mental health outcomes, but the results help contextualize the extent of internalized stigmas that may be experienced by MSW. Despite the small sample size, however, rich narratives from 60–90-minute interviews allowed for a deeper understanding of the psychological impact of sociolegal interventions.

Notably, all participants were low-income. Studies exploring maternal mental health and broader maternal health needs among sex workers should account for varying pathways to motherhood, compositions of family, and working structures. This will further elucidate how unique occupational conditions based on the sectors of sex work participants engage in may expose them to different legal vulnerabilities and subsequent maternal health outcomes. The sexual commerce industry is not a monolith. It is a highly variable industry and sex workers are a heterogenous group on the basis of identity, economic status, and parenting desires. The participants in this study engaged in many forms of sex work and experienced many forms of motherhood. Research that is reflective of the diversity within the industry will lead to responsive and localized policy solutions.

Many sex workers co-parent (34); however, a majority of the parents in the present sample were not co-parenting. Further, many sex workers who are parents co-provide care with a partner. The majority of participants in our study were not partnered and if they were co-providing care, were co-providing care with another family member, such as a parent. Future studies can build on these limitations by exploring experiences across a variety of family structures and considering how different family members are impacted by discriminatory socio-legal interventions.

### Future directions

While MSW must navigate marginalization, there may also be an opportunity to explore novel mothering and caregiving strategies that are positively informed by their sex-working role. For example, MSW may be skilled at promoting body positivity and discussing the importance of consent and agency with their children. MSW often report great pride in their maternal roles and strong desire to be a mother (2, 3). A positive maternal identity for sex workers whose parenthood is central to their sense of self may be a protective factor against the harms of socio-legal interventions on their well-being, improving mental health and wellbeing. Exploring matrescence among MSW highlights opportunities to focus on the assets of MSW during this life transition and also may identify disruptors that can be controlled or prevented.

## Conclusion

The understanding of matrescence, or development of maternal identity, is still under-explored among MSW, yet our study findings support the centrality of mothering to MSW. Additionally, our findings highlight the pain of institutional and structural forces that impose family separation, divert women from health care contact, and force MSW into situations in which their feelings and family needs go unmet. The same structural violence that those involved in sex work are vulnerable to is present here and perhaps exacerbated by the vulnerability of having given birth, needing to care for children (desperate fatigue, for example), and the mental worry of having children seized by CPS. Such worries are confirmed by intermittent experiences that are not acknowledged as traumatic by the CPS, a system that is focused on child welfare and criminalizes substance use. While these events are distressing, further work is needed to disambiguate mental health issues such as postpartum depression, and the ostensibly normal reaction of distress to stressful and traumatic events, which may in turn lead to disrupted maternal development or matrescence. This study exemplifies the utility of matrescence as a framework to highlight the omnipresence of carcerality on the lives of sex-working parents.

## Data Availability

Due to the small sample size which contains rich narratives of a systems-marginalized population, data are not available. Requests to access the datasets should be directed to rmoss3@tulane.edu.
